# Hypoxia-Inducible Factor-1*α* in Macrophages, but Not in Neutrophils, Is Important for Host Defense during *Klebsiella pneumoniae*-Induced Pneumosepsis

**DOI:** 10.1155/2021/9958281

**Published:** 2021-08-05

**Authors:** Natasja A. Otto, Liza Pereverzeva, Valentine Leopold, Ivan Ramirez-Moral, Joris J. T. H. Roelofs, Jeroen W. J. van Heijst, Alex F. de Vos, Tom van der Poll

**Affiliations:** ^1^Center for Experimental and Molecular Medicine, Amsterdam University Medical Centers, University of Amsterdam, Amsterdam, Netherlands; ^2^Amsterdam Infection & Immunity Institute, Amsterdam, Netherlands; ^3^Department of Pathology, Amsterdam University Medical Centers, University of Amsterdam, Amsterdam, Netherlands; ^4^Neogene Therapeutics, Amsterdam, Netherlands; ^5^Division of Infectious Diseases, Amsterdam University Medical Centers, University of Amsterdam, Amsterdam, Netherlands

## Abstract

Hypoxia-inducible factor- (HIF-) 1*α* has been implicated in the ability of cells to adapt to alterations in oxygen levels. Bacterial stimuli can induce HIF1*α* in immune cells, including those of myeloid origin. We here determined the role of myeloid cell HIF1*α* in the host response during pneumonia and sepsis caused by the common human pathogen *Klebsiella pneumoniae*. To this end, we generated mice deficient for HIF1*α* in myeloid cells (LysM-cre × *Hif1α*^fl/fl^) or neutrophils (Mrp8-cre × *Hif1α*^fl/fl^) and infected these with *Klebsiella pneumoniae* via the airways. Myeloid, but not neutrophil, HIF1*α*-deficient mice had increased bacterial loads in the lungs and distant organs after infection as compared to control mice, pointing at a role for HIF1*α* in macrophages. Myeloid HIF1*α*-deficient mice did not show increased bacterial growth after intravenous infection, suggesting that their phenotype during pneumonia was mediated by lung macrophages. Alveolar and lung interstitial macrophages from LysM-cre × *Hif1α*^fl/fl^ mice produced lower amounts of the immune enhancing cytokine tumor necrosis factor upon stimulation with *Klebsiella*, while their capacity to phagocytose or to produce reactive oxygen species was unaltered. Alveolar macrophages did not upregulate glycolysis in response to lipopolysaccharide, irrespective of HIF1*α* presence. These data suggest a role for HIF1*α* expressed in lung macrophages in protective innate immunity during pneumonia caused by a common bacterial pathogen.

## 1. Introduction

Sepsis is a complex syndrome characterized by a dysregulated host response to an infection resulting in organ dysfunction and associated with a high mortality risk [1]. Sepsis is a major global health problem with an estimated 48.9 million incident cases recorded worldwide and 11 million sepsis-related deaths in 2017, representing a fifth of all global deaths that year [2]. The pathobiology of sepsis is poorly understood, which together with the heterogeneity of this syndrome has been held responsible for the failure of clinical trials seeking to establish sepsis-specific immune modulatory therapies. The majority of sepsis cases (54-64%) originate from pneumonia [3, 4], and *Klebsiella* (*K.*) *pneumoniae* is a common causative pathogen in pneumonia and sepsis [5, 6]. The relevance of *K. pneumoniae*-induced infections is further indicated by the emergence of antibiotic-resistant strains.

Cellular metabolism plays an important role in immune cell function [7]. Cells with different immunological functions use distinct metabolic pathways to generate the required amount of energy and biosynthetic intermediates for proliferation and/or protein synthesis. Generally, proinflammatory responses are associated with a shift towards glycolysis (the breakdown of glucose to pyruvate) while an anti-inflammatory profile is linked with energy generation through the tricarboxylic acid (TCA) cycle and oxidative phosphorylation (OXPHOS). Glycolysis is a relatively inefficient pathway in terms of energy yield, but it provides the cell with many biosynthetic intermediates to support anabolic growth. Macrophages were reported to alter their metabolic profile in response to lipopolysaccharide (LPS), a proinflammatory component of the gram-negative bacterial cell wall, in a way that depended on their source [8]. Bone marrow-derived macrophages (BMDMs) stimulated with LPS responded with a profound upregulation of glycolysis and downregulation of OXPHOS, while peritoneal macrophages showed upregulation of both glycolysis and OXPHOS. In addition, whole bacteria may modify energy metabolism in a way that differs from effects induced by purified bacterial components [9]. This suggests that energy metabolism in myeloid cells may vary depending on the site of infection and bacterial stimulus.

Hypoxia-inducible factor-1 (HIF1) is a key regulator of glycolysis. HIF1 consists of two subunits, HIF1*α* and HIF1*β*, with the latter being endogenously present in cells. HIF1*α* is constitutively synthesized but, when oxygen is present, rapidly hydroxylased by prolyl hydroxylase (PHD) 2, marking it for degradation by the ubiquitin-proteasome pathway [10]. Under hypoxic conditions, the lack of oxygen inactivates PHD2 resulting in the stabilization of HIF1*α*. Upon dimerization, HIF1 translocates to the nucleus where it induces the transcription of genes encoding proteins that enhance glucose transport, glycolysis, and the conversion of pyruvate into lactate instead of entering the TCA cycle [11]. In immune cells, HIF1*α* can also be stabilized by oxygen-independent mechanisms. Macrophages contain increased HIF1*α* levels upon exposure to different pathogens [12], and activation with LPS induces HIF1*α* expression in a NF-*κ*B-dependent manner [13, 14], suggesting a role for HIF1*α* during macrophage activation. Indeed, peritoneal macrophages lacking HIF1*α* showed decreased glycolysis and tumor necrosis factor (TNF) secretion, and HIF1*α*-deficient BMDMs demonstrated less intracellular killing capacity in vitro [15]. However, the role of myeloid cell HIF1*α* in the host response during bacterial pneumonia and pneumosepsis is unexplored and not easy to predict considering that the metabolic programming of macrophages depends on their subtype/origin [8, 16]. Therefore, we here aimed to study the role of myeloid cell HIF1*α* in the host defense during pneumonia-derived sepsis using a well-established model via low-dose infection with *K. pneumoniae* via the airways [17–19], resulting in a gradually growing bacterial load in the lungs with subsequent dissemination and sepsis, allowing analyses of both early protective and late injurious responses associated with innate immune activation.

## 2. Materials and Methods

### 2.1. Animals

Homozygous *Hif1α*^fl/fl^ mice (007561, Jackson Laboratory) [20] were crossed with LysM-cre [21] or Mrp8-cre mice (021614, Jackson Laboratory) [22] to generate myeloid- (LysM-cre × *Hif1α*^fl/fl^) and neutrophil- (Mrp8-cre × *Hif1α*^fl/fl^) specific Hif1*α*-deficient mice, respectively [23]. *Hif1α*^fl/fl^ Cre-negative littermates were used as controls in all experiments. All genetically modified mice were backcrossed at least six times to a C57Bl/6 background. Mice were age and sex matched and used in experiments at 8-12 weeks of age. Studies involving animals were reviewed and approved by the Central Authority for Scientific Procedures on Animals (CCD) and the Animal Welfare Body (IvD) Institutional Animal Care and Use Committee of the Academic Medical Center (AMC), University of Amsterdam (identification numbers 17-4125-1-04 and -50). The animal care and use protocol adhered to the Dutch Experiments on Animals Act (WOD) and European Directive of 22 September 2010 (Directive 2010/63/EU) in addition to the Directive of 6 May 2009 (Directive 2009/41/EC).

### 2.2. Cell Stimulation

Naïve mice were anesthetized with isoflurane and then sacrificed by cervical dislocation. Alveolar macrophages (AMs) were harvested by bronchoalveolar lavage (BAL) with PBS containing 2 mM EDTA. Cells were seeded in 96-well flat-bottom culture plates (Greiner Bio-One) at a density of approximately 40,000 cells per well in RPMI complete media (containing 10% FBS, penicillin/streptomycin, 2 mM L-glutamine, and 25 mM HEPES; Gibco) and left to adhere overnight. AMs were stimulated for 24 hours with 100 ng/ml ultrapure LPS (*E. coli* O111: B4; InvivoGen) or medium control.

### 2.3. Western Blot

AMs were treated with 50 *μ*M IOX2 (inhibitor of PHD2; HY-15468, MedChemExpress) for 24 hours to stabilize HIF1*α* protein and lysed in RIPA buffer (150 mM NaCl, 1% Triton X-100, 0.5% sodium deoxycholate, 0.1% SDS, and 50 mM Tris HCl, pH 8) supplemented with HALT protease and phosphatase inhibitor (Thermo Fisher) and stored at -20°C until processing. Samples were resolved in Laemmli buffer (0.1875 M Tris HCl, pH 6.8, 6% SDS, 10% *β*-mercaptoethanol, 30% glycerol, and 0.006% bromophenol blue) and heated for 5 min at 95°C. Samples were loaded on 10% polyacrylamide precast gels (Bio-Rad) and transferred to PVDF membranes. After incubation for 1 hour with blocking buffer at room temperature, immunoblotting was performed using rabbit anti-HIF1*α* (14179) and rabbit anti-*β*-Actin (4967 L; both Cell Signaling). A goat anti-rabbit antibody (7074S; Cell Signaling) conjugated with horseradish peroxidase was used as a secondary antibody. Blots were incubated with the Lumi-Light detection kit (Roche), and pictures were taken using ImageQuant LAS-4000 (GE Healthcare).

### 2.4. Mouse Infection Models

Pneumonia was induced by intranasal inoculation with approximately 10,000 colony forming units (CFU) of *K. pneumoniae* serotype 2 (ATCC 43816; American Type Culture Collection) as described [17–19]. After 12 or 40 hours of infection, mice were anesthetized by injection with ketamine/medetomidine and sacrificed by cardiac puncture followed by cervical dislocation. In a separate experiment, mice were infected with *K. pneumoniae* (~1∗10^4^ CFU) intravenously via the tail vein as described [17, 24] and euthanized after 36 hours. Whole lungs, spleens, and livers were harvested and partly fixed in formalin and partly homogenized in four volumes of sterile saline with a tissue homogenizer (ProScience, Oxford, CT). Bacterial loads in the lung, blood, spleen, and liver were determined by counting CFU from serial dilutions plated on blood agar plates, incubated at 37°C for 16 hours. For cytokine and chemokine measurements, lung homogenates were lysed in an equal volume of lysis buffer (150 mM NaCl,15 mM Tris, 1 mM MgCl, 1 mM CaCl2, and 1% Triton, pH 7.4) with protease inhibitors (Roche Complete Protease Inhibitor cocktail) on ice for 30 min and spun down. Supernatants were stored for analysis.

### 2.5. Assays

Interleukin- (IL-) 1*β*, IL-10, IL-6, and tumor necrosis factor- (TNF-) *α* were measured by ELISA according to the manufacturer's protocol (R&D Systems, Minneapolis, MN). Lactate was quantified using an enzymatic assay, as described before [9]. Briefly, lactate was oxidized by lactate oxidase, and the resulting H_2_O_2_ was coupled to the conversion of the Amplex Red reagent to fluorescent resorufin by horseradish peroxidase. Samples were diluted 200 times and incubated for 20 minutes. Fluorescence was measured using a 96-well plate reader (BioTek, Winooski, VT).

### 2.6. Histopathology and Immunohistochemistry

The lung, spleen, and liver were fixed in 10% formaldehyde and embedded in paraffin. Four-micrometer sections of the lung were stained with hematoxylin and eosin (H&E) and scored by an independent pathologist as described [17, 18]. The following parameters were scored on a scale of 0 (absent), 1 (mild), 2 (moderate), 3 (severe), and 4 (very severe): interstitial damage, vasculitis, peribronchitis, oedema, thrombus formation, and pleuritis. In all experiments, the samples were scored by the same pathologist blinded for experimental groups. Neutrophil influx was determined by immunohistochemical staining with the Ly-6G monoclonal antibody (mAb; clone 1A8; BioLegend, San Diego, CA). Slides were scanned with the Philips IntelliSite Ultra Fast Scanner 1.6RA (Philips Digital Pathology Solutions, Best, The Netherlands), and TIFF images, spanning the full tissue section, were generated. In these images, Ly-6G positivity and total surface area were measured using ImageJ (version 2006.02.01, U.S. National Institutes of Health, Bethesda, MD); the amount of Ly-6G positivity was expressed as the percentage of the total surface area.

### 2.7. Lung Digestion and Flow Cytometry

Lung digestion and flow cytometry were done in essence as described [19]. Briefly, the lungs were washed in PBS, minced into pieces, and incubated at 37°C for 30 minutes with warm PBS containing 10 mg/ml DNase I (Roche) and 5 mg/ml Liberase TM (Sigma-Aldrich). Cells were filtered, washed several times with PBS, seeded at a density of approximately 1 × 10^6^ cells per well in RPMI complete media, and stimulated for 2.5 hours with heat-killed *K. pneumoniae* (K. pneu) or left untreated. To study intracellular TNF, cells were treated with a protein transport inhibitor (containing brefeldin A; BD Biosciences). To study glucose uptake and mitochondrial mass in lung suspensions, cells were incubated for 3 hours with the addition of 50 *μ*g/ml 2-(N-(7-nitrobenz-2-oxa-1,3-diazol-4-yl)amino)-2-deoxyglucose (2NBDG; Cayman Chemical; Ann Arbor, MI) or 50 nM MitoTracker Green (Invitrogen) during the last 30 minutes of incubation. Reactive oxygen species (ROS) production was measured by stimulating lung cell suspensions with heat-killed *K. pneumoniae* or heat-killed *Candida albicans* (C. albicans; UC820, generously provided by Dr. Leo Joosten, Radboud UMC, Nijmegen, the Netherlands) with the addition of 10 *μ*M carboxy-H_2_DCFDA (Invitrogen) during the last 30 minutes of culture. Phagocytosis was analyzed by incubation with 250 *μ*g/ml pHrodo™ Red *E. coli* BioParticles™ Conjugate (Invitrogen). Cell subsets were identified by staining with fixable viability dye eFluor 780 (Invitrogen) and the following antibodies: rat anti-mouse CD16/CD32 (clone 93), rat anti-mouse CD45 PE-eFluor610 (clone 30-F11), rat anti-mouse CD11b PE-Cy7 (clone M1/70), rat anti-mouse Siglec-F Alexa Fluor 647 (clone E50-2440), and rat anti-mouse Ly-6G Alexa Fluor 700 (clone 1A8) (all from BD Biosciences) and mouse anti-mouse CD64 PerCP-Cy5,5 (clone X54-5/7.1) and rat anti-mouse MerTK PE (clone 2B10C42) (all from BioLegend, San Diego, CA). Intracellular staining with rat anti-mouse TNF Alexa Fluor 488 (clone MP6-XT22; BioLegend) was performed using Foxp3/Transcription Factor Staining Buffer Set (eBioscience, San Diego, CA). Flow cytometry was performed using a FACSCanto II (BD Biosciences), and data were analyzed using FlowJo software (BD Biosciences).

### 2.8. Statistical Analysis

Nonparametric variables were analyzed using the Mann-Whitney *U* test. Parametric variables were analyzed using Student's *t*-tests (2-group comparison) or a 2-way ANOVA (comparison between 3 or more groups) with Sidak's multiple comparison test where appropriate. Analysis was done using GraphPad Prism version 8 (GraphPad Software, San Diego, CA). Statistical significance is shown as ^∗^*P* < 0.05, ^∗∗^*P* < 0.01, ^∗∗∗^*P* < 0.001, and ^∗∗∗∗^*P* < 0.0001.

## 3. Results

### 3.1. Alveolar Macrophages from LysM-cre × *Hif1α*^fl/fl^ Mice Are HIF1*α* Deficient and Produce Less TNF and IL-6 upon LPS Stimulation In Vitro

**Figure 1:**
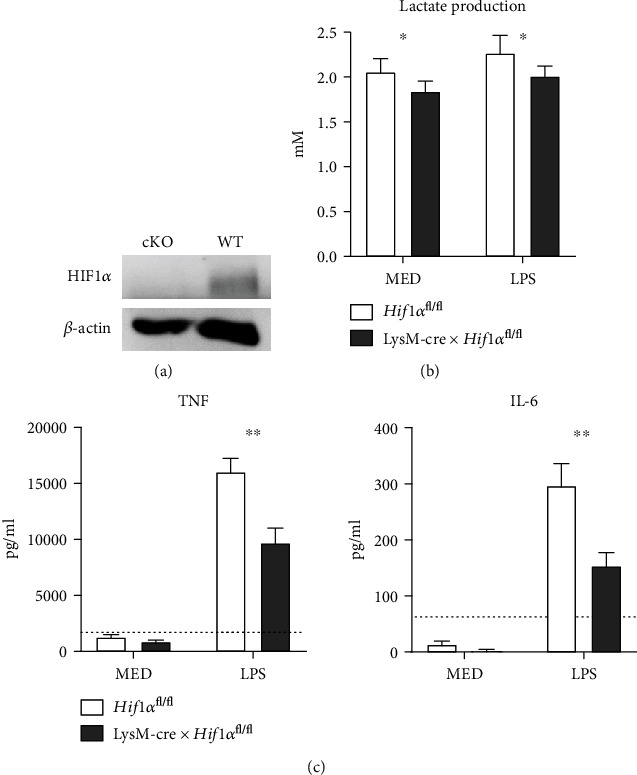
HIF1*α* is important for glucose metabolism and TNF production of alveolar macrophages. HIF1*α* protein expression in AMs derived via BAL from naïve LysM-cre × *Hif1α*^fl/fl^ mice (cKO) and littermate controls (WT) treated with IOX2 for 24 hours (a). Lactate (b) and cytokine (TNF and IL-6) production (c) by AMs stimulated in vitro with LPS or left untreated for 24 hours. Data are shown as bar graphs showing mean with standard error of the mean from 6 technical replicates of pooled AMs from 7 mice per group. Dotted line indicates the reliable lower limit of detection of the cytokine assays. Lactate production by LysM-cre × *Hif1α*^fl/fl^ AMs was compared to that of control AMs (*Hif1α*^fl/fl^) of mice using *t*-tests. TNF and IL-6 production of LPS-stimulated cKO AMs and control AMs was compared using the Mann-Whitney test. ^∗^*P* < 0.05; ^∗∗^*P* < 0.01.

In order to document successful deletion of *Hif1α* in AMs from LysM-cre × *Hif1α*^fl/fl^ mice, we harvested AMs from BAL fluid and cultured these in the presence of the PHD2 inhibitor IOX2. Inhibition of PHD2 results in stabilization of HIF1*α* thereby allowing detection of the protein, which otherwise is rapidly degraded [10, 25]. Western blotting detected HIF1*α* in IOX2-treated AMs from *Hif1α*^fl/fl^ (control) mice but not from LysM-cre × *Hif1α*^fl/fl^ mice (Figure 1(a)). Exposure of either HIF1*α*-deficient or control AMs to LPS did not result in lactate release into the medium, suggesting that AMs do not mount a glycolytic response to this gram-negative bacterial cell wall component (Figure 1(b)). Of interest, however, HIF1*α*-deficient AMs consistently released less lactate than control AMs, irrespective of the presence of LPS. HIF1*α*-deficient AMs produced less TNF and IL-6 than control AMs upon LPS stimulation (Figure 1(c)); IL-1*β* and IL-10 production was not detectable by either HIF1*α*-deficient or control AMs.

### 3.2. Macrophage HIF1*α* Is Important for Host Defense during *Klebsiella pneumoniae*-Induced Pneumosepsis

**Figure 2:**
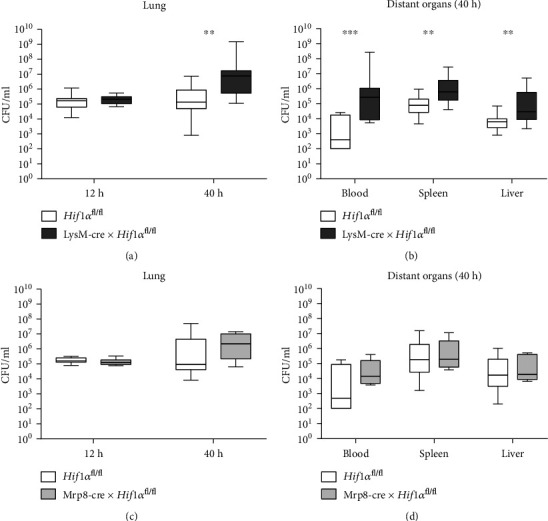
Macrophage HIF1*α* is important for host defense against *K. pneumoniae* in the lung. Bacterial loads (CFU/ml) in the lungs of LysM-cre × *Hif1α*^fl/fl^ mice and littermate controls 12 and 40 hours after intranasal inoculation with ~10^4^ CFU *K. pneumoniae* (a) or in distant organs 40 hours after inoculation (b). Data are shown as box-and-whisker diagrams of 13-16 mice per group from two independent experiments for each time point. Bacterial loads (CFU/ml) in the lungs of Mrp8-cre × *Hif1α*^fl/fl^ mice and littermate controls 12 and 40 hours after intranasal inoculation with ~10^4^ CFU *K. pneumoniae* (c) or in distant organs 40 hours after inoculation (d). Data are shown as box-and-whisker diagrams of 7-8 mice per group at each time point. Bacterial loads of the LysM-cre × *Hif1α*^fl/fl^ mice were compared to those of littermate control (*Hif1α*^fl/fl^) mice using the Mann-Whitney test. ^∗^*P* < 0.05; ^∗∗^*P* < 0.01; ^∗∗∗^*P* < 0.001.

To determine the importance of HIF1*α* in macrophages during pneumonia-induced sepsis, we assessed the bacterial outgrowth and dissemination of intranasally instilled *K. pneumoniae* during pneumonia (12 hours after inoculation) and pneumosepsis (40 hours after inoculation) in LysM-cre × *Hif1α*^fl/fl^ mice and littermate (*Hif1α*^fl/fl^) controls. HIF1*α* deficiency in myeloid cells did not affect bacterial outgrowth within 12 hours of infection. However, after 40 hours of infection, bacterial loads in the lungs (Figure 2(a)) as well as dissemination to distant organs (blood, spleen, and liver; Figure 2(b)) were increased in LysM-cre × *Hif1α*^fl/fl^ mice relative to *Hif1α*^fl/fl^ control mice. Since Cre expression driven by the LysM promoter also occurs to a certain extent in neutrophils [15, 23], we examined a possible role for neutrophil HIF1*α* by generating neutrophil-specific HIF1*α*-deficient mice (Mrp8-cre × *Hif1α*^fl/fl^ mice) [23]. Bacterial outgrowth in the lung and dissemination to distant organs were similar in Mrp8-cre × *Hif1α*^fl/fl^ and to *Hif1α*^fl/fl^ control mice (Figures 2(c) and 2(d)). To determine whether the increased bacterial loads in the distant organs of LysM-cre × *Hif1α*^fl/fl^ mice were due to impaired host defense locally or caused by the increase in bacterial loads in the lung, we injected *K. pneumoniae* intravenously in LysM-cre × *Hif1α*^fl/fl^ mice and *Hif1α*^fl/fl^ control mice. Both mouse strains had similar bacterial loads in the lung, blood, spleen, and liver 36 hours after intravenous infection (Supplemental Figure S1). The finding that intranasal inoculation but not intravenous injection of *K. pneumoniae* results in different bacterial loads in the lung suggests that HIF1*α* in alveolar macrophages (AMs) rather than interstitial macrophages (IMs) is important for host defense in this organ. Together, these results suggest that HIF1*α* in alveolar macrophages, but not in neutrophils, is important for host defense against pneumonia-derived sepsis caused by *K. pneumoniae*.

### 3.3. Macrophage HIF1*α* Deficiency Is Associated with Higher Cytokine Levels in the Lung Early after Induction of Pneumonia

**Figure 3:**
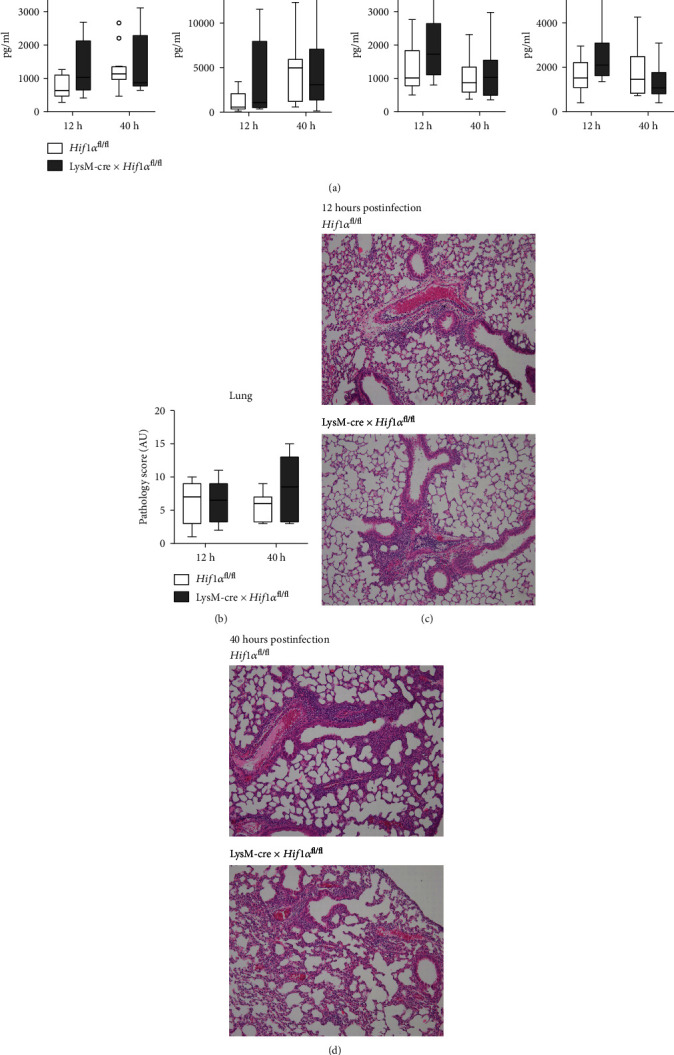
Pathology of the lungs after *K. pneumoniae* infection. Cytokine levels (TNF, IL-1*β*, IL-6, and IL-10) in the lungs of 12-16 mice per group 12 and 40 hours after infection with *K. pneumoniae* (a). The extent of inflammation in the lungs of LysM-cre × *Hif1α*^fl/fl^ mice and littermate controls scored on haematoxylin and eosin- (H&E-) stained tissue sections as total pathology score of the infected lungs from 7-8 mice per group at 12 and 40 hours after inoculation (b). Data are shown as box-and-whisker diagrams, and the LysM-cre × *Hif1α*^fl/fl^ mice were compared to littermate control (*Hif1α*^fl/fl^) mice using the Mann-Whitney test. Representative photographs of H&E-stained tissue sections of the infected lungs from LysM-cre × *Hif1α*^fl/fl^ mice and littermate controls at 12 (c) and 40 hours (d).

To obtain insight into the role of macrophage HIF1*α* in the induction and perpetuation of lung inflammation during *Klebsiella* pneumonia, we determined the extent of lung pathology, neutrophil influx, and pulmonary cytokine levels. Remarkably, we found higher TNF, IL-1*β*, IL-6, and IL-10 in lung homogenates of LysM-cre × *Hif1α*^fl/fl^ mice when compared with *Hif1α*^fl/fl^ control mice at 12 hours after inoculation; these differences were not present anymore at 40 hours after infection (Figure 3(a)). The degree and characteristics of lung pathology, as determined by H&E staining scored by an independent pathologist blinded for experimental groups, were similar between LysM-cre × *Hif1α*^fl/fl^ mice and littermate controls (Figures 3(b) – 3(d)). Likewise, neutrophil influx, determined by quantification of positive Ly-6G staining and measurements of MPO in whole lung homogenates, did not differ between mouse strains (Supplemental Figure S2).

### 3.4. Lung Macrophages from LysM-cre × *Hif1α*^fl/fl^ Mice Produce Less TNF upon Stimulation of Whole Lung Cell Suspensions with *K. pneumoniae*

**Figure 4:**
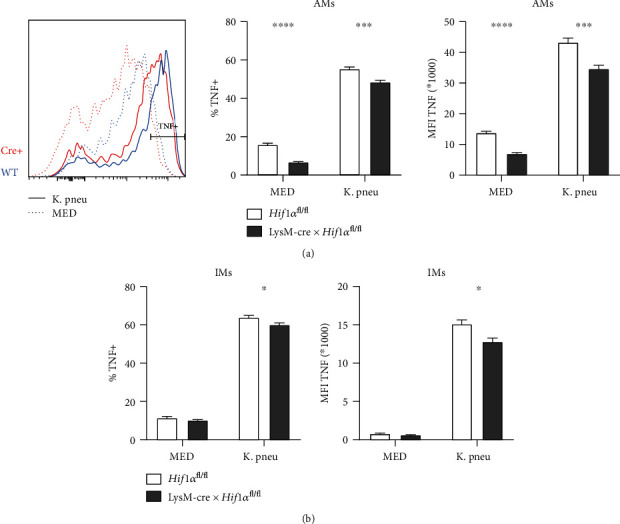
HIF1*α* deficiency affects TNF production capacity of AMs and IMs. Percentage TNF-positive cells and the mean fluorescent intensity (MFI) of the TNF staining of AMs (a) and IMs (b) from LysM-cre × *Hif1α*^fl/fl^ (Cre+) and control mice (WT) after stimulation of lung suspensions for 2.5 hours with heat-killed *K. pneumoniae* (K. pneu) or medium control (MED). Data are shown as bar graphs showing mean with standard error of the mean from 10 mice per group. MFIs and percentage positive cells were compared using Student's *t*-tests followed by the Holm-Sidak multiple comparison test. ^∗^*P* < 0.05; ^∗∗^*P* < 0.01; ^∗∗∗^*P* < 0.001; ^∗∗∗∗^*P* < 0.0001.

TNF plays a pivotal role in host defense during *K. pneumoniae* pneumonia [26–28]. The discrepancy between the results obtained with AMs from LysM-cre × *Hif1α*^fl/fl^ mice (reduced TNF production upon LPS stimulation in vitro, Figure 1) and LysM-cre × *Hif1α*^fl/fl^ mice after infection with viable *K. pneumoniae* via the airways (higher TNF levels in whole lung homogenates at 12 hours after infection, Figure 3(a)) prompted us to study macrophage-specific TNF production in whole lung cell suspensions exposed to heat-killed *K. pneumoniae*. To this end, we used intracellular TNF staining followed by flow cytometry to determine the capacity of AMs (SiglecF^high^, CD11b^neg^) and interstitial macrophages (IMs; SiglecF^neg^, CD11b^high^) from LysM-cre × *Hif1α*^fl/fl^ and *Hif1α*^fl/fl^ control mice to produce TNF, expressing this as the percentage TNF-positive (%TNF+) cells and median cell fluorescence intensity (MFI) (Figure 4). Incubation with *K. pneumoniae* induced a strong increase in the %TNF+ and TNF MFI of AMs and IMs of both LysM-cre × *Hif1α*^fl/fl^ and control mice. Importantly, AMs from LysM-cre × *Hif1α*^fl/fl^ mice displayed a strongly reduced capacity to produce TNF in response to *K. pneumoniae*; diminished intracellular TNF staining of AMs from LysM-cre × *Hif1α*^fl/fl^ mice was already present in unstimulated lung cell suspensions. IMs from LysM-cre × *Hif1α*^fl/fl^ mice also produced less TNF after exposure of lung cell suspensions to *K. pneumoniae*, although the difference with control IMs was not as large as for AMs. The phagocytic capacity of AMs and IMs was determined by incubation with pHrodo Red *E. coli* BioParticles™. While IMs showed a higher phagocytic capacity than AMs (as shown by a higher percentage of positive cells and higher MFIs), differences in the HIF1*α* genotype had no effect (Supplemental Figure S3A-B). Finally, we determined the capacity of AMs and IMs to produce ROS; in these experiments, we exposed lung cell suspensions not only to *K. pneumoniae* but also to *C. albicans* considering its potency to induce ROS [29] (Supplemental Figure S3B-C). Indeed, while *K. pneumoniae* did not induce ROS in AMs or IMs, *C. albicans* elicited a marked increase in ROS in both macrophage subsets. However, again differences in the HIF1*α* genotype had no effect.

### 3.5. Lung Macrophages from LysM-cre × *Hif1α*^fl/fl^ Mice Take Up Less Glucose

**Figure 5:**
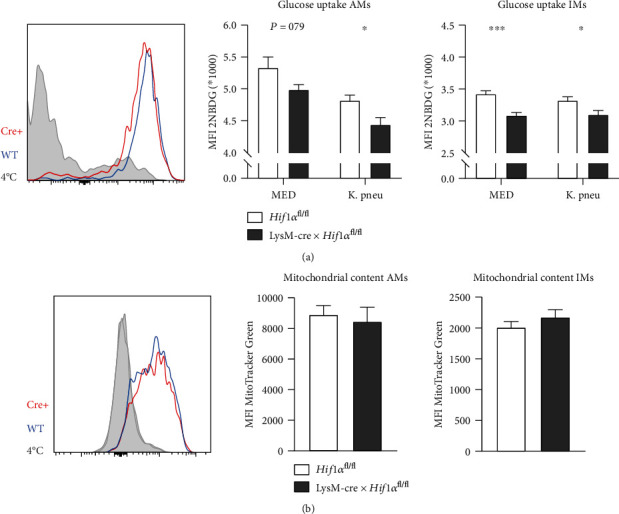
HIF1*α* deficiency affects glucose metabolism but not mitochondrial mass of AMs and IMs. Glucose uptake after 2.5 hours of stimulation with heat-killed *K. pneumoniae* (K. pneu) or medium control (MED), as measured by the MFI of 2NBDG, of AMs and IMs within lung suspensions of LysM-cre × *Hif1α*^fl/fl^ mice and littermate controls (a). Mitochondrial mass as measured by the MFI of the MitoTracker Green probe in AMs and IMs of LysM-cre × *Hif1α*^fl/fl^ mice and littermate control after 3 hours of incubation in medium (b). As a negative control, lung suspensions were kept at 4°C. Data are shown as bar graphs showing mean with standard error of the mean of 10 mice per group for the glucose uptake assay and 6 mice per group for the mitochondrial mass. Groups were compared using Student's *t*-tests with the Holm-Sidak multiple comparison test where appropriate. ^∗^*P* < 0.05; ^∗∗^*P* < 0.01; ^∗∗∗^*P* < 0.001.

To determine the effect of HIF1*α* deficiency on glucose metabolism of AMs and IMs, whole lung cell suspensions were incubated with exogenously added 2NBDG, a fluorescent analog of glucose, or MitoTracker Green probe. Incubation of lung cell suspensions with *K. pneumoniae* was not associated with increased glucose uptake by either AMs or IMs (Figure 5(a)). However, AMs and IMs from LysM-cre × *Hif1α*^fl/fl^ mice took up less 2NBDG when compared with control macrophages, in both unstimulated and *Klebsiella*-stimulated conditions. Mitochondrial staining by MitoTracker Green showed no difference in mitochondrial mass in AMs and IMs from LysM-cre × *Hif1α*^fl/fl^ and control mice (Figure 5(b)).

## 4. Discussion

HIF1*α* has been studied extensively as an orchestrator of the cellular response to low oxygen [11]. In the context of infection, HIF1*α* can be induced due to the hypoxic environment of inflamed tissue and through stimulation of cells with bacterial components [30]. Myeloid cell HIF1*α* has been implicated in the regulation of cellular energy metabolism as well as immune responses and may play a role in host defense against infection [30]. Here, we sought to determine the role of myeloid HIF1*α* in the host response during pneumonia and sepsis caused by *K. pneumoniae*, a common gram-negative human pathogen. To this end, we generated mice with myeloid cell-specific deficiency of HIF1*α* and infected these with a virulent strain of *K. pneumoniae* via the airways. Mice with myeloid but not with neutrophil HIF1*α* deficiency demonstrated an impaired defense as reflected by increased bacterial growth in the lungs and enhanced dissemination to distant organs. Myeloid cell HIF1*α*-deficient mice did not show increased bacterial burdens after intravenous infection, suggesting a protective role for HIF1*α* in lung macrophages. Both AMs and IMs from myeloid cell HIF1*α*-deficient mice produced less TNF upon exposure to *K. pneumoniae*, which considering the central role of TNF in host defense against this bacterium [26, 28, 31] could at least in part explain the more vulnerable phenotype of myeloid HIF1*α*-deficient mice.

Our finding that HIF1*α*-deficient macrophages produced less TNF in vitro is corroborated by earlier studies. Peritoneal macrophages from LysM-cre × *Hif1α*^fl/fl^ mice showed an approximate 25% reduction in TNF release upon LPS exposure [15], and bone marrow-derived macrophages from LysM-cre × *Hif1α*^fl/fl^ mice produced less TNF upon stimulation with group A streptococci [12]. Our study expands these data to AMs and lung IMs. In agreement, LysM-cre × *Hif1α*^fl/fl^ mice demonstrated reduced release of TNF-*α* after intraperitoneal LPS administration, which was associated with a strongly improved survival [32]. The immune enhancing effect of local TNF, expressed in the lungs, during pneumonia caused by *Klebsiella* has been demonstrated in several ways: treatment with various anti-TNF strategies [26, 31] and genetic deletion of the gene encoding TNF or TNF receptor type I [28] resulted in increased bacterial loads during *Klebsiella* pneumonia, and conversely, intrapulmonary delivery of a TNF agonist peptide augmented host defense after infection with *K. pneumoniae* via the airways [33]. Together, these data suggest that the reduced macrophage-associated TNF production in the lungs of LysM-cre × *Hif1α*^fl/fl^ mice contributed to the enhanced bacterial growth and dissemination in these animals. TNF levels in whole lung homogenates of LysM-cre × *Hif1α*^fl/fl^ mice were higher than those in control mice at 12 hours after infection. This enhanced TNF response in whole lungs did not impact bacterial loads and likely was derived from TNF-producing cells other than AMs and IMs. In this respect, it should be noted that Cre-recombinase driven by the LysM promoter is primarily expressed in macrophages and neutrophils, while less so or not at all in monocytes, dendritic cells, lymphoid cells, and parenchymal cells [21, 23, 34]. Our study is limited by the fact that we did not identify cellular sources of TNF in the lungs other than AMs and IMs.

Remarkably, we found higher TNF, IL-1*β*, IL-6, and IL-10 in lung homogenates of LysM-cre × *Hif1α*^fl/fl^ mice when compared with *Hif1α*^fl/fl^ control mice at 12 hours after inoculation, but these differences in pulmonary cytokine levels were not present after 40 hours of infection. The model of *Klebsiella*-induced pneumonia used here is associated with a gradually growing bacterial load accompanied by steadily increasing proinflammatory cytokine levels, which is highly dependent on bacterial numbers [17, 18, 35]. Therefore, it is surprising to find higher cytokine levels in the lungs of LysM-cre × *Hif1α*^fl/fl^ mice when compared with *Hif1α*^fl/fl^ control mice at 12 hours after inoculation, since the bacterial loads were similar at this point. Even more surprising is the finding that at 40 hours of infection, when the bacterial loads in the lungs of LysM-cre × *Hif1α*^fl/fl^ mice were higher than those of littermate controls, lung cytokine levels were not higher anymore, suggesting a bimodal effect of myeloid HIF1*α* on cytokine production in the lungs (inhibitory early after infection while—relatively—enhancing later on, during fulminant sepsis). Interestingly, elevated HIF1*α* levels have been linked with IRAK-M-induced immune suppression in monocytes [36] which would support an immunosuppressive effect of HIF1*α*. Conversely, HIF1 pathway activation has also been associated with extended effector responses and inhibiting “exhaustion” of CD8+ T cells [37]. Furthermore, glycolysis-dependent peritoneal macrophages lacking HIF1*α* showed impaired motility, TNF production, and bacterial killing due to a drastically reduced ATP pool as a result of inhibited glycolysis [15]. Proinflammatory responses generated in immune cells are usually associated with enhanced cellular glycolysis, which provides a fast energy source. Several macrophage subtypes show a glycolytic response to stimulation with LPS, including BMDMs and peritoneal macrophages [38]. We here demonstrate that AMs from either LysM-cre × *Hif1α*^fl/fl^ or control mice do not mount a glycolytic response upon stimulation with LPS, as indicated by unaltered lactate release relative to medium control conditions. This result is in agreement with recent reports from our and other laboratories that murine AMs do not enhance glycolysis in response to LPS [19, 39]. Nonetheless, HIF1*α*-deficient AMs released less lactate than wild-type AMs irrespective of the presence of LPS, suggesting that HIF1*α* does regulate the constitutive glycolytic state of these cells. Under homeostatic conditions, AMs have oxygen readily available for the production of energy to sustain their functions. However, it is possible that in highly inflamed lungs, oxygen availability is impaired and HIF1*α* deficiency might impair AM functions at a later stage of the infection. These data illustrate the complexity of the role of immunometabolism in host defense, where the tissue environment of immune cells can impact the specifics of the metabolic changes directing inflammatory reactions [8, 16].

Besides in macrophages, LysM-cre × *Hif1α*^fl/fl^ mice show extensive deletion of *Hif1α* in neutrophils [15], which is in agreement with the cellular distribution of LysM expression in reporter mice [23]. In order to discriminate between myeloid- and neutrophil-specific roles of HIF1*α*, we generated Mrp8-cre × *Hif1α*^fl/fl^ mice, thereby making use of the almost exclusively neutrophil-restricted expression of the Mrp8 promoter [23]. Mrp8-cre × *Hif1α*^fl/fl^ mice showed an unaltered antibacterial defense, arguing against a role for HIF1*α* in neutrophils during *Klebsiella* pneumonia. HIF1*α* has been implicated in NET formation by neutrophils [40], but whether NETs impact the response to *Klebsiella* is unknown.

The capacity of lung macrophages from LysM-cre × *Hif1α*^fl/fl^ mice to phagocytose and to produce ROS was not altered when compared to lung macrophages from control mice. In previous studies, bone marrow-derived macrophages from LysM-cre × *Hif1α*^fl/fl^ mice showed an impaired capacity to kill group A streptococci and *Pseudomonas aeruginosa* [12], and inhibition of HIF1*α* in neutrophils resulted in diminished killing of *Pseudomonas* [41]. The highly virulent *Klebsiella* strain used in the current experiments cannot be killed by wild-type immune cells in vitro (Ref [42] and data not shown), precluding bacterial killing assays with macrophages from LysM-cre × *Hif1α*^fl/fl^ mice. Our finding of impaired antibacterial defense in LysM-cre × *Hif1α*^fl/fl^ mice is corroborated by a study reporting the importance of myeloid HIF1*α* for limiting the systemic spread of bacteria during skin infection by group A streptococci [12]. Moreover, in a model of keratitis induced by *Pseudomonas aeruginosa*, silencing of HIF1*α* led to increased bacterial growth [41]. Of note, respiratory epithelial HIF1*α* has been shown to limit bacterial dissemination to the spleen during *Klebsiella* pneumonia, while it was not required for the induction of cytokines and chemokines in the airways [43].

We here report that HIF1*α* deficiency in myeloid cells results in enhanced bacterial growth in pneumonia and sepsis caused by *K. pneumoniae*. We further show that this phenotype likely is caused by HIF1*α* deficiency in lung macrophages and associated with a reduced capacity of these cells to produce the immune enhancing cytokine TNF. These data suggest a role for macrophage HIF1*α* in protective innate immunity during infection caused by a common bacterial pathogen.

## Data Availability

Data are available on request to the corresponding author.

## Supplementary Materials

Supplementary Materials Supplemental Figure S1: HIF1*α* is not important for host defense after intravenous injection of *K. pneumoniae*. Supplemental Figure S2: myeloid HIF1*α* deficiency does not affect neutrophil influx nor MPO production in the lung. Supplemental Figure S3: HIF1*α* deficiency does not affect phagocytosis and ROS production by AMs and IMs.
