# Supplementary material for: Hypoxia-Inducible Factor-1α in Macrophages, but Not in Neutrophils, Is Important for Host Defense during Klebsiella pneumoniae-Induced Pneumosepsis
Source: Mediators Inflamm. 2021 Aug 5;2021:9958281. doi: 10.1155/2021/9958281 (PMC8360744; doi:10.1155/2021/9958281)
Supplement: Supplementary Materials — Supplemental Figure S1: HIF1α is not important for host defense after intravenous injection of K. pneumoniae. Supplemental Figure S2: myeloid HIF1α deficiency does not affect neutrophil influx nor MPO production in the lung. Supplemental Figure S3: HIF1α deficiency does not affect phagocytosis and ROS production by AMs and IMs. [file 9958281.f1.docx]

**Supplementary Descriptions**

**Supplemental Figure S1: HIF1α is not important for host defense after intravenous injection of *K. pneumoniae***

Bacterial loads (CFU/ml) in the lung, blood, spleen and liver of LysM-cre x *Hif1α*^fl/fl^ mice and littermate controls 36 hours after intravenous inoculation with ~10^4^ CFU *K. pneumoniae.* Data are shown as box-and-whisker diagrams of 8-9 mice per group. Bacterial loads of the LysM-cre x *Hif1α*^fl/fl^ mice were compared to littermate control (*Hif1α*^fl/fl^) mice using the Mann-Whitney test.

**Supplemental Figure S2: Myeloid HIF1α-deficiency does not affect neutrophil influx nor MPO production in the lung**

Neutrophil accumulation in lung tissue of LysM-cre x *Hif1α*^fl/fl^ mice and littermate controls 12 and 40 hours after intranasal inoculation with ~10^4^ CFU *K. pneumoniae* measured by Ly-6G staining and quantified as the percentage of Ly-6G stained area of lung tissue (A). Myeloperoxidase (MPO) concentrations in lung homogenates of LysM-cre x *Hif1α*^fl/fl^ mice and littermate controls 12 and 40 hours after intranasal inoculation with ~10^4^ CFU *K. pneumoniae* (B). Groups were compared using Student t-tests with the Holm-Sidak’s multiple comparisons test.

**Supplemental Figure S3: HIF1α-deficiency does not affect phagocytosis and ROS production by AMs and IMs**

Percentage positive cells and the mean fluorescent intensity (MFI) of the phagocytosed *E.coli* particles^TM^ of AMs (A) and IMs (B) from LysM-cre x *Hif1α*^fl/fl^ and control mice after incubation of lung suspensions with *E.coli* particles^TM^ for 2,5 hours. Percentage positive cells and the mean fluorescent intensity (MFI) of ROS production of AMs (C) and IMs (D) from LysM-cre x *Hif1α*^fl/fl^ and control mice after stimulation of lung suspensions with heat-killed *K. pneumoniae* (K. pneu), heat-killed *C. albicans* (C. alb) or medium control for 2,5 hours with addition of Carboxy-H_2_DCFDA the last 30 minutes of culture. As a negative control lung suspensions were kept at 4°C. Data are shown as bar graphs showing mean with standard error of the mean from 6 mice per group. MFI’s and percentage positive cells were compared using Student t-tests followed by the Holm-Sidak’s multiple comparisons test when appropriate.

**Supplementary Materials**

**Supplemental Figure S1: HIF1α is not important for host defense after intravenous injection of *K. pneumoniae***

Bacterial loads (CFU/ml) in the lung, blood, spleen and liver of LysM-cre x *Hif1α*^fl/fl^ mice and littermate controls 36 hours after intravenous inoculation with ~10^4^ CFU *K. pneumoniae.* Data are shown as box-and-whisker diagrams of 8-9 mice per group. Bacterial loads of the LysM-cre x *Hif1α*^fl/fl^ mice were compared to littermate control (*Hif1α*^fl/fl^) mice using the Mann-Whitney test.

**Supplemental Figure S2: Myeloid HIF1α-deficiency does not affect neutrophil influx nor MPO production in the lung**

Neutrophil accumulation in lung tissue of LysM-cre x *Hif1α*^fl/fl^ mice and littermate controls 12 and 40 hours after intranasal inoculation with ~10^4^ CFU *K. pneumoniae* measured by Ly-6G staining and quantified as the percentage of Ly-6G stained area of lung tissue (A). Myeloperoxidase (MPO) concentrations in lung homogenates of LysM-cre x *Hif1α*^fl/fl^ mice and littermate controls 12 and 40 hours after intranasal inoculation with ~10^4^ CFU *K. pneumoniae* (B). Groups were compared using Student t-tests with the Holm-Sidak’s multiple comparisons test.

**Supplemental Figure S3: HIF1α-deficiency does not affect phagocytosis and ROS production by AMs and IMs**

Percentage positive cells and the mean fluorescent intensity (MFI) of the phagocytosed *E.coli* particles^TM^ of AMs (A) and IMs (B) from LysM-cre x *Hif1α*^fl/fl^ and control mice after incubation of lung suspensions with *E.coli* particles^TM^ for 2,5 hours. Percentage positive cells and the mean fluorescent intensity (MFI) of ROS production of AMs (C) and IMs (D) from LysM-cre x *Hif1α*^fl/fl^ and control mice after stimulation of lung suspensions with heat-killed *K. pneumoniae* (K. pneu), heat-killed *C. albicans* (C. alb) or medium control for 2,5 hours with addition of Carboxy-H_2_DCFDA the last 30 minutes of culture. As a negative control lung suspensions were kept at 4°C. Data are shown as bar graphs showing mean with standard error of the mean from 6 mice per group. MFI’s and percentage positive cells were compared using Student t-tests followed by the Holm-Sidak’s multiple comparisons test when appropriate.
